# Progress in Xenotransplantation: Immunologic Barriers, Advances in Gene Editing, and Successful Tolerance Induction Strategies in Pig-To-Primate Transplantation

**DOI:** 10.3389/fimmu.2022.899657

**Published:** 2022-05-18

**Authors:** Daniel L. Eisenson, Yu Hisadome, Kazuhiko Yamada

**Affiliations:** ^1^ Columbia Center for Translational Immunology, Department of Medicine, Columbia University Irving Medical Center, New York, NY, United States; ^2^ Department of Surgery, The Johns Hopkins Hospital, Baltimore, MD, United States; ^3^ Department of Surgery, Columbia University Irving Medical Center, New York, NY, United States

**Keywords:** xenotransplantation, thymic transplantation, Mixed hematopoietic cell chimerism, thymokidney, vascularized thymic lobe transplantation

## Abstract

Organ transplantation is the most effective treatment for end stage organ failure, but there are not enough organs to meet burgeoning demand. One potential solution to this organ shortage is xenotransplantation using pig tissues. Decades of progress in xenotransplantation, accelerated by the development of rapid genome editing tools, particularly the advent of CRISPR-Cas9 gene editing technologies, have enabled remarkable advances in kidney and heart xenotransplantation in pig-to-nonhuman primates. These breakthroughs in large animal preclinical models laid the foundation for three recent pig-to-human transplants by three different groups: two kidney xenografts in brain dead recipients deemed ineligible for transplant, and one heart xenograft in the first clinical grade study of pig-to-human transplantation. However, despite tremendous progress, recent data including the first clinical case suggest that gene-modification alone will not overcome all xenogeneic immunologic barriers, and thus an active and innovative immunologic strategy is required for successful xenotransplantation. This review highlights xenogeneic immunologic barriers, advances in gene editing, and tolerance-inducing strategies in pig-to-human xenotransplantation.

## Introduction

Almost seventy years after the first successful kidney transplant by Joseph Murray in 1954 (1), transplantation has become standard of care for end organ failure. Murray’s transplant was termed homotransplantation, as it occurred between genetically identical twins; success in allotransplantation, or transplant between genetically different humans, required advances in immunosuppression that soon came with azathioprine and later cyclosporine (2, 3). As a result of this success, one of the central problems in the field of transplantation is that there are simply not enough organs to meet demand. Xenotransplantation, or transplant across species barriers, has long been proposed to solve this organ shortage, but interspecies immunologic barriers proved difficult to overcome (4–7).

Early xenotransplantation efforts utilized nonhuman primates (NHPs) as donors and achieved some notable successes. In 1963-1964, Keith Reemtsma at Tulane University in Louisiana transplanted chimpanzee kidneys into six patients with end stage renal failure (without long-term dialysis options), utilizing immunosuppression of azathioprine, actinomycin C, and steroids. While most recipients rejected organs within eight weeks of transplant, one patient survived for nine months (8). Due to ethical concerns about the use of primates in terminal procedures as well as practical issues of organ availability, the xenotransplantation community shifted from primates to pigs as donors (9–11). Pigs are more widely available, but present greater immunologic barriers: much of xenotransplantation research for the last thirty years has been devoted to understanding the immunologic obstacles in pig-to-primate transplantation and to modifying both donor pigs as well as recipient NHP immunologic responses.

In addition to immunologic barriers limiting organ survival in pig-to-primate transplantation, concerns about the transmission of zoonotic infections tempered early enthusiasm for xenotransplantation. These concerns were heightened with the 1997 discovery of porcine endogenous retroviruses (PERVs) that could infect human epithelial cells *in vitro* (12), and catalyzed additional research in PERVs – some groups have used genome editing to inactivate PERVs in pigs used for xenotransplantation (13) – as well as the development of diagnostic tools to assess for other pig-derived pathogens, including porcine cytomegalovirus (PCMV) (14). While comprehensive discussion of zoonosis is outside the scope of this review, pre-clinical and early clinical data suggest that zoonotic infection from swine will likely be rare – there has been no evidence for infections of humans or nonhuman primates caused by pig xenotransplantation products (14).

Decades of progress in xenotransplantation, accelerated by the development of rapid genome editing technology, particularly the advent of CRISPR-Cas9 technologies (15, 16), have enabled remarkable advances in pig-to-NHP transplantation: >6 months survival in life-supporting pig-to-baboon kidney transplants (17, 18), >6 months survival of heterotopic and orthotopic pig-to-baboon heart models (19–21), and, more recently >1 year survival in life-supporting pig-to-macaque kidney transplants (22). These breakthroughs in large animal preclinical models laid the foundation for clinical experiments in the United States. Over the last six months, three institutions have transitioned from preclinical studies using NHP recipients to preclinical studies (and one clinical study) using human recipients. Each of these approaches was different. New York University (NYU) and the University of Alabama (UAB) implanted kidneys into brain dead patients (deemed ineligible for organ donation) for a short period of time (less than 74 hours). The NYU team used kidneys from pigs that were genetically engineered to remove α-1,3-galactosyltransferase, called Gal “knock out” (GalTKO) pigs, while the UAB team used kidneys from pigs that were the product of more extensive gene editing: four genes “knocked out” (including α-1,3-galactosyltransferase, and pig growth hormone receptor), and multiple human genes (including genes encoding complement regulatory proteins and other proteins involved in normal human anti-coagulation) added. The University of Maryland Medical Center (UMMC) identified a patient with heart failure in the absence of liver or kidney failure who was not a candidate for implanted mechanical circulatory support or allotransplant and obtained FDA approval for compassionate use of a xenograft. They used the same genetically modified pigs as those used in the UAB study.

These recent experiments confirmed encouraging results from NHP studies and have led to a resurgence of enthusiasm for xenotransplantation. However, the UMMC clinical case also suggests that additional strategies will be required for long-term success of clinical xenotransplantation. Gene-modification alone cannot overcome all xenogeneic immunologic barriers, and an active and innovative immunologic strategy will be required for successful xenotransplantation. Considering these developments and the imminent future of clinical xenotransplantation, this article provides a brief overview of immunologic barriers in pig-to-human xenotransplantation, highlights progress achieved through gene editing technologies, and outlines a way forward through tolerance-inducing strategies.

## Immunological Barriers in Pig-To-Primate Xenotransplantation

Transplanted organs across species barriers elicit more robust immune responses than are seen in allotransplantation. The innate immune system plays a more active role in rejection of xenografts than it does in rejection of allografts for reasons that we will detail, but both the innate and adaptive immune systems participate in these responses.

### A. Innate Immune Barriers to Xenotransplantation

The key components of the innate immune system that are involved in rejection of xenografts are natural antibodies, complement systems, and macrophages/natural killer cells.

Natural antibodies (Nabs) directed against carbohydrate antigens on pig cells presented one of the first and most important obstacles to pig-to-primate xenotransplantation. These preformed antibodies – categorized as innate immunity because they are present without specific exposure to pig cells – led to hyperacute rejection within hours of transplantation. Galili et al. discovered and isolated one important subset of these antibodies that is directed against a carbohydrate component (α-1,3-galactose, or “α-gal”) of a cell surface glycoprotein produced by an enzyme (α-1,3-galactosyltransferase) that is not functional in humans or old-world primates (23). Discovery of anti-gal antibodies in humans that recognize α-1,3-galactose residues on pig cells precipitated a decades-long effort to produce pigs without α-1,3-galactosyltransferase (24–27). Two additional targets of Nabs, NeuGc, a glycoprotein produced by cytidine-monophosphate-N-acetyl-neuraminic acid hydroxylase (CMAH) which is inactive in humans (28), and SDa, a blood group antigen produced by porcine β-1,4-N-acetyl-galactosaminyltransferase 2 (β4GALNT2), have also been identified (29). Together, antibodies to these three carbohydrate antigens make up more than 95% of preformed antibodies against pig cells (30, 31).

Incompatibilities between pig and primate complement systems were also recognized to be a significant barrier to pig-to-primate xenotransplantation. Complement proteins, activated by Nab-binding (classical pathway) or spontaneous binding (alternative pathway), have also been shown to lead to vascular injury and rejection, as porcine complement regulatory proteins do not effectively inhibit human complement activation (32). Accordingly, pigs expressing human complement regulatory proteins were some of the first transgenic pigs made for the purpose of xenotransplantation (33).

Macrophages and natural killer cells, too, have been shown to play important roles in rejection of pig-to-primate xenografts. Both cell types are present in rejecting xenografts, and studies suggest that these cells may generate potent anti-graft responses independent of T-cell activity (34, 35). Macrophages participate in rejection of grafts both indirectly, as mediators of inflammation, and directly, phagocytosing cells. Phagocytosis of xenogeneic cells has been the subject of particular study: signal regulatory protein (SIRP)α, an inhibitory receptor on macrophages that binds to CD47 on human cells to prevent phagocytosis, does not respond to porcine CD47 (36). As will be discussed later in this review, this discovery of inhibitory signaling species incompatibilities between pigs and primates has made human CD47 (hCD47) a candidate transgene for further genetic modifications of pig used in xenotransplantation.

### B. Adaptive Immune Barriers to Xenotransplantation

As detailed above, the innate immune system presents formidable species-specific barriers to xenotransplantation that are not seen in human-to-human transplantation. In allotransplantation, the innate immune system plays a minor role, but the adaptive immune responses are primarily responsible for rejection of grafts. This disconnect led many to hypothesize that the innate immune system was the critical barrier to xenotransplantation and that adaptive responses were less important.

It was initially believed that differences between pig and primate MHC proteins would inhibit effective MHC-binding and adaptive immune responses across species barriers. Early studies in a pig-to-mouse transplant model seemed to confirm this theory, suggesting that xenogeneic adaptive immune responses were weaker than allogeneic immune responses (37); however, this was later found to be a function of decreased costimulatory signaling between mice and pigs, and not related to TCR-MHC binding (38). Indeed, subsequent studies have shown that human T cells are directly activated by antigens exposed by porcine SLA molecules, that they respond to xeno-MHC antigens at least as well as they do to allo-MHC antigens (39) *in vitro*, and that costimulatory interactions between porcine MHC and human MHC molecules are not limited by species incompatibilities as in a pig-to-mouse transplant models (40) *in vivo*.

In addition to *direct* human TCR and pig SLA interactions, human B and T cells are activated *indirectly via* presentation of pig antigens on human antigen presenting cells (41). In this way, B cells presenting pig antigens are induced by cognate T cells to produce anti-non gal antibodies (42). These antibodies precipitate antibody-mediated rejection, which, as in humans, is difficult to control with immunosuppressive medications. Given the large proportion of pig proteins that are slightly different from their functional equivalents in humans, there are countless other possible xenoantigens that may trigger adaptive induced antibody production. While the innate immune system presents an early barrier to survival of pig-to-human xenografts, overcoming adaptive immune responses – in particular, induced antibody production – will be essential for long-term survival of pig xenografts.

## Genetic Engineering for Pig-To-Primate Transplantation

As discussed earlier, hyperacute rejection in early pig-to-primate transplants – due, in large part, to pre-formed Nabs – limited enthusiasm for the use of pigs as organ donors. Isolation and identification of these Nabs and their dominant target, α-1,3-galactose, led to a race to eliminate α-1,3-galactosyltransferase (Gal) from pig genomes. Once these genetically modified GalTKO pigs were available in 2003, the senior author of this review performed the first life-supporting GalTKO pig-to-baboon kidney transplant. Hyperacute rejection was successfully avoided in this case, and baboon survival was extended further – from 29 days with hCD55 grafts (43) to 83 days (44) – when combining these genetically modified kidneys with tolerance strategies (discussed later). Since this initial breakthrough, the scientific community has made remarkable recent strides in our ability to modify the pig genome to create organs suitable for transplant in humans.

### A. Early Application of Gene Editing Technologies in Xenotransplantation

Before GalTKO pigs were available, researchers used various techniques to deplete preformed Nabs, including plasmapheresis, immunoadsorption columns, and use of a xenograft to act as a sponge (i.e. transplanting one kidney to absorb antibodies, and then removing it and replacing it with a second kidney) (45). These methods achieved some success, especially when used in combination with the first transgenic pigs, which were created using recombinant DNA plasmid embryo microinjection and expressed human complement regulatory proteins CD59 (Fodor, PNAS, 1994) and then CD55 (33, 46). However, Nabs returned after initial depletion and prevented long-term graft survival.

In 2002 and 2003, two groups created the first GalTKO pigs using somatic cell nuclear transfer technology, and provided a major breakthrough in the field of pig-to-primate transplantation (25–27, 47). Subsequent preclinical studies demonstrated that transplantation of heart or kidney xenografts using GalTKO pigs were significantly less likely to undergo hyperacute rejection (44, 48). Instead, these grafts – with the exception of those that were co-transplanted with thymus tissue – were rejected after weeks to months, and rejection was correlated with elicited anti-non gal antibodies.

### B. CRISPR-Cas9 and Current Targets of Gene Modification

Because xenografts using GalTKO pigs were still ultimately rejected, researchers sought to eliminate newly revealed targets of anti-non-gal Nabs, and to further modify these knockout animals with additional human transgenes. This process was slow, and multiply modified pigs proved difficult to produce. However, discovery of CRISPR-Cas9 facilitated rapid genome manipulation and led to creation of animals – as were used by UMMC and UAB – with ten genetic modifications (16).

The first category genetic manipulation involves removal of genes to eliminate targets of Nabs. In addition to α-1,3-galactose (carbohydrate produced by α-1,3-galactosyltransferase), two other Nab targets were discovered, including Neu5Gc (produced by cytidine-monophosphate- N-acetyl-neuraminic acid hydroxylase, or CMAH, inactivated in humans), and SDa (produced by beta‐1,4‐N‐acetyl‐galactosaminyltransferase 2, or β4GALNT2) (30, 31, 49, 50). Triple knockout pigs (created without Gal, CMAH, and β4GALNT2) demonstrate markedly reduced Nab-binding *in vitro* and may confer a survival advantage when combined with other transgenes in pig-to-cynomolgus macaques transplant model (51). It is worth noting that the true impact of some genetic modifications may be difficult to evaluate in a NHP model, as in the case of CMAH knockout. Old world primates have a functional CMAH gene and so do not have Nabs that bind to anti-Neu5Gc; paradoxically, inactivation of CMAH gene increased NHP antibody binding when compared to GalTKO alone, which suggests that inactivation of CMAH may reveal new epitopes and present new targets for Nab-binding (52).

The second major category of genetic modification involves insertion of human transgenes to correct for dysregulation in complement, coagulation, and inflammatory pathways due to species incompatibilities. As mentioned above, the first genetically modified pigs created for xenotransplantation included human complement regulatory proteins CD55 (33, 46) and later iterations included CD46 (53). Coagulation regulatory proteins including human thrombomodulin (hTBM) and endothelial protein receptor C (EPCR) were also early targets of genetic modification (54). More recent targets include anti-inflammatory proteins like heme-oxygenase (HO-1) (55, 56) and anti-phagocytic proteins, including human macrophage inhibitory ligand CD47 (57). With the advent of CRISPR-Cas9 gene editing technologies, it is now possible to combine these genetic manipulations and create multiply modified animals.

### C. Limits of Gene Modification for Xenotransplantation

This extraordinary progress has led some to speculate that xenotransplantation will ultimately be accomplished through ever increasing genetic modification. This may be part of the answer, but there are three key issues with this approach. First, the xenotransplant community remains divided over exactly which genetic modifications are necessary. While some modifications, including GalTKO, are well-studied, others are supported only by mouse models or by a small number of cases in NHP models. Cost is a major obstacle to definitive research here, as genetically modified animals are expensive to produce, and transplants in NHPs are expensive to conduct. However, these modifications may have unintended consequences (e.g. creation of neoepitopes, seen in transplant of CMAH KO pigs to old world monkeys) (58), and it is important that each individual modification is subject to rigorous scrutiny.

Second, while genes may be reliably incorporated within the genome using new gene editing technologies, uneven gene expression in transgenic animals remains an issue. Of particular concern is tissue-specific gene expression. For example, transgenes that are only expressed in the liver will not be effective in kidney xenotransplantation. On a more granular level, studies have shown that human CD47 expression in pig endothelial cells and podocytes prevented phagocytosis by correcting CD47-SIRPα species incompatibilities and enabling normal SIRPα signal transduction. Indeed, hCD47 expression on podocytes in hCD47/GalTKO pig-to-baboon kidney transplants was shown to inhibit the development of proteinuria (59).. However, our data also demonstrated that high hCD47 expression in renal tubular cells may lead to destructive inflammatory responses *via* the hCD47-TSP-1 pathway (59). More research is needed to further refine and reliably predict gene expression in multiply modified pigs.

Third, while genetic modifications may help protect pig xenografts from innate responses, genetic engineering alone is not sufficient to prevent rejection long-term, given the overwhelming number of possible xenoantigens that could trigger adaptive responses and antibody-mediated rejection. Accordingly, additional strategies such as targeted immunosuppression or, as will be discussed in the following section, tolerance induction of B and T cells, will be required for long-term survival or xenografts.

## Strategies to Promote Tolerance in Pig-To-Human Transplantation

Tolerance-inducing approaches have achieved donor-specific tolerance of renal grafts in allotransplantation and have been under investigation for use in xenotransplantation for over twenty-five years. One of the first hurdles in applying this strategy in pig-to-human transplantation was theoretical: MHC interactions and costimulatory signaling are essential for the development of tolerance, and there was concern that these interactions would be limited by species incompatibilities (37). However, subsequent studies demonstrated effective interspecies adaptive immune interactions and dispelled initial skepticism about the ability of human and pig T cells to communicate effectively (39, 60). Eventually, tolerance induction may allow for reduction or cessation of immunosuppression; in the near-term, these tolerance strategies may serve as a critical adjunct to immunosuppression to overcome xenogeneic barriers. The specific strategies addressed by this review include 1) thymic transplantation and 2) mixed hematopoietic cell chimerism.

### A. Vascularized Thymic Transplantation

Transplantation of donor thymus has proven to be a potent strategy for tolerance induction. While this method is less studied in clinical allotransplantation than mixed hematopoietic cell chimerism, thymic transplantation has emerged as most effective tolerance strategy for xenotransplantation currently under investigation.

Early studies from Sykes et al. demonstrated that transplantation of porcine thymic tissue in thymectomized mice resulted in production of mature mouse T cells that were tolerant of porcine skin grafts *via* intrathymic deletion of donor reactive T cells (61, 62). These studies helped to resolve concerns about the efficacy of MHC interactions across xenogeneic barriers and paved the way for subsequent large animal studies. However, non-vascularized pig thymic grafts did not survive long enough to engraft or to promote tolerance in early large animal studies – grafts were rejected and recipient pigs found to have anti-donor elicited antibodies even across allogeneic barriers in a pig-to-pig model (63).

**Figure 1:**
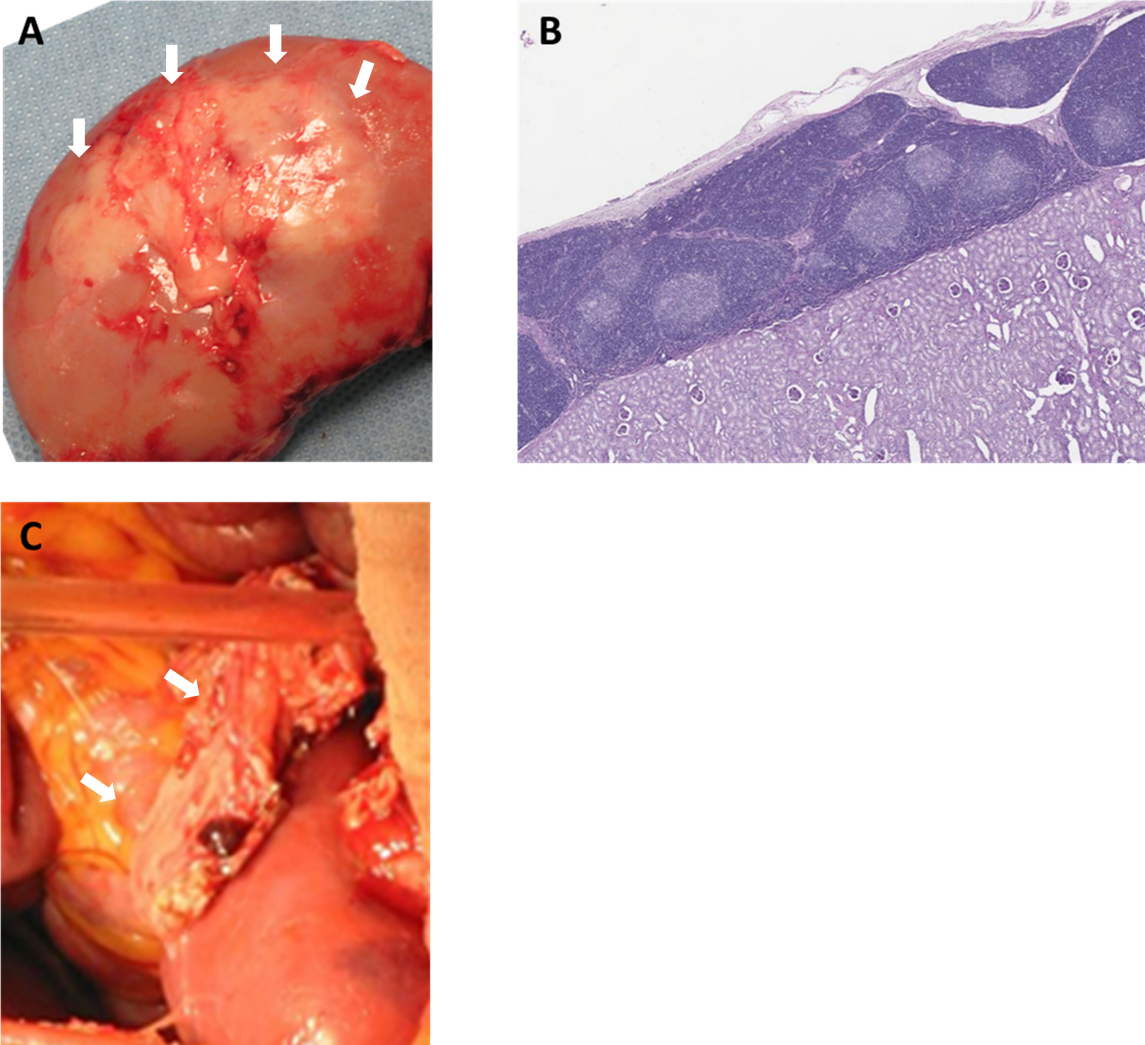
Vascularized donor thymic graft transplantation. **(A)** Porcine composite “thymokidney” at 5 weeks after thymic tissue implantation under own renal capsule, and **(B)** histology of the thymokidney (HE). **(C)** Co-transplantation of porcine vascularized thymic lobe and kidney graft in a baboon. White arrows indicate thymic grafts.

To prevent rejection of these ischemic thymic grafts, the senior author of this review developed two methods to transplant vascularized thymic grafts: (1) composite thymus+kidney (“thymokidney”) transplant (**Figures 1A, B**) (64); and (2) vascularized thymic lobe transplant (**Figure 1C**) (65) in the late 1990s and early 2000s. These vascularized thymic grafts proved able to participate immediately in the induction of tolerance and supported thymopoiesis across allogeneic swine kidney and heart transplant models (66–70). While these gains in pig-to-primate transplantation were modest in the era before GalTKO pigs, likely due to robust Nab binding, the results were striking when using organs from the first GalTKO pigs: thymus co-transplantation with GalTKO kidneys prolonged survival of recipient baboons from 29 days (43) to 83 days with donor-specific unresponsiveness (44). While this strategy successfully prevented sensitization of recipient baboons to the xenograft, all baboon recipients suffered serious complication of nephrotic proteinuria. Despite relatively preserved renal function and renal histology showing only minimal absence of anti-pig antibody (Ab) deposits by immunofluorescence, proteinuria was observed as early as post-operative day (POD) 2 (71). Over the past 5 years, we have developed strategies to prevent proteinuria, combining novel therapeutic agents (including CTLA4-Ig) with additional genetic engineering (hCD47) (18, 71, 72). With these new regimens, we achieved long-term survival of >6 months in multiple recipients of vascularized thymus plus kidneys (18, 59). While hCD47 may be helpful, it is worth noting that we were able to avoid early development of proteinuria and achieve 193 days of rejection free survival using kidney+thymus from GalTKO pig without further genetic modification (18). Given uncertainty about which genetic modifications are necessary and the unintended consequences of additional modifications, “single-gene” kidneys (GalTKO alone) – combined with our vascularized thymic graft to induce tolerance – may represent the best path forward in recipients with low levels of anti-pig non Gal preformed NAb. The NYU group elected to use thymokidneys in their pig-to-human kidney transplants in brain dead patients, September-November, 2021 (NYTimes, Oct 21st, 2021).

Both types of vascularized thymic grafts appear to be effective at inducing tolerance of co-transplanted donor kidneys across xenogeneic barriers (18, 44, 59), but there are advantages and disadvantages to each method. Thymokidneys (64) may be more feasible to create and thus more broadly applicable for kidney xenotransplantation; vascularized thymic lobe (VTL) (65) harvest is technically challenging and prone to ischemia due to convoluted blood supply. However, in contrast to the thymokidney, VTL may theoretically be used to induce tolerance of any other organ graft (70). Additional studies will compare these methods of thymic graft creation, will combine thymic transplantation with mixed chimerism tolerance strategies, and will explore the role of thymus co-transplantation in tolerance induction of other solid organs.

### B. Mixed Hematopoietic Cell Chimerism

Mixed bone marrow chimerism – in which a recipient produces both donor and recipient (self) hematopoietic cells through hematopoietic stem cell transplantation (HSCTx) after non-myeloablative conditioning (73–75) – has been shown to induce tolerance across HLA barriers in multiple clinical studies (76–78). Early studies in pig-to-mouse transplant models suggested that mixed chimerism was possible with non-myeloablative conditioning regimens (79), and that this strategy could induce tolerance across xenogeneic barriers in a rodent model. Critically, mixed chimerism in mice was found to induce tolerance to the α-gal epitope, demonstrating tolerance of B cells with disappearance of natural antibodies (80). Thus, not only did mixed chimerism prevent hyperacute rejection in rodent models, but it also prevented T-cell mediated rejection and antibody-mediated rejection (81) in a pig-to-mouse model.

However, mixed chimerism has proven more difficult to achieve in preclinical pig-to-primate transplant models. Attempts of GalTKO pig HSCTx in baboons resulted in loss of bone marrow cells within 24 to 48 hours (82, 83). This rapid elimination of xenogeneic cells is likely due to innate immune mechanisms: primate macrophages have been shown to phagocytose porcine cells independent of antibody or complement binding (84) and may be related to species incompatibilities between porcine macrophage inhibitory ligand CD47 and human macrophage SIRPα which result in failure of anti-phagocytosis signaling (85). As mentioned earlier, one strategy to prevent this problem of early phagocytosis is to create transgenic pigs expressing hCD47. In a pig-to-baboon skin transplant model, hematopoietic cells from transgenic pigs expressing hCD47 lasted longer than cells from pigs with porcine CD47 after HSCTx; accordingly, skin grafts from hCD47 pigs demonstrated improved survival when compared to grafts from CD47 wild type pigs (86). In this way, genetic engineering may be used to facilitate tolerance strategies by allowing for the establishment of mixed chimerism.

Another strategy to avoid rapid consumption of porcine hematopoietic stem cells after HSCTx and promote bone marrow engraftment is to inject porcine bone marrow cells directly into the bone. The senior author of this review developed the intra-bone bone marrow (IBBM) transplant procedure in a pig-to-baboon model. This technique prolonged peripheral macrochimerism for 3 weeks and enabled engraftment of porcine hematopoietic progenitors in four of six baboon recipients of GalTKO pigs (87). Moreover, enhanced mixed chimerism after IBBM Tx resulted in transient anti-pig unresponsiveness that translated to prolonged survival of GalTKO kidneys after subsequent kidney transplant. Notably, by combining these methods, using hCD47-GalTKO pigs and IBBM Tx, we have achieved >60 days macrochimerism associated with reduction in anti-pig IgG (88). Despite initial setbacks, mixed chimerism remains a promising approach for induction of tolerance across xenogeneic barriers.

Although vascularized thymic transplant can induce T cell tolerance and inhibit T cell-mediated B cell and NK cell responses across xenogeneic barriers, B cells and NK cells are not “tolerized” by thymic transplantation in a pig-to-baboon model. Additional gene knockouts may limit rejection of pig organs by non-Gal Nabs, but run the risk of exposing new, currently unidentified, antigenic targets and/or compromising the health of the pig. Accordingly, we are currently working on combining vascularized thymus and IBBM Tx strategies to establish robust long-term T and B cell tolerance in xenotransplantation.

## Conclusion

Once a researcher’s pipe dream, xenotransplantation is now becoming a clinical reality. After many years of progress, catalyzed by advances in gene editing technologies, three groups have confirmed decades of NHP research and established the short-term viability of pig-to-human transplantation. All three pig-to-human experiments demonstrated that hyperacute rejection (NYU, UAB, and UMMC) can be avoided through use of genetically modified pigs and immunosuppression. UMMC’s pig-to-human heart transplant represents a landmark accomplishment and the start of a new era in clinical xenotransplantation. Despite the success of this critical initial step, the UMMC patient’s clinical course should also serve to galvanize the application of innovative strategies, such as tolerance induction, to ensure long-term survival of pig xenografts.

## Author Contributions

DE: Primarily in writing the review paper. YH: Participated in data analysis of the cited references. KY: Corresponding author. Primarily outlines and finalizes the review paper. All authors contributed to the article and approved the submitted version.

## Funding

This research was partially supported by NIH grant, P01AI45897 and UO1 AI152881.

## Conflict of Interest

The authors declare that the research was conducted in the absence of any commercial or financial relationships that could be construed as a potential conflict of interest.

## Publisher’s Note

All claims expressed in this article are solely those of the authors and do not necessarily represent those of their affiliated organizations, or those of the publisher, the editors and the reviewers. Any product that may be evaluated in this article, or claim that may be made by its manufacturer, is not guaranteed or endorsed by the publisher.

## Abbreviations

β4GALNT2, β-1,4-N-acetyl-galactosaminyltransferase 2; CD40L, CD40 ligand; CMAH, cytidine-monophosphate-N-acetyl-neuraminic acid hydroxylase; EC, endothelial cell; EPCR, endothelial protein receptor; GalTKO, α-1,3 Galactosyl transferase gene knockout; hCD47, human CD47; HO, heme-oxygenase; HSCTx. Hematopoietic stem cell transplantation; hTBM, human thrombomodulin; IBBMTx, intra-bone bone marrow transplantation; MHC, major histocompatibility complex; mAb, monoclonal antibody; Nab, natural antibody; NHP, non human primate; NK, natural killer; NYU, New York University; PBMC, Peripheral blood mononuclear cells; POD, post operative day; SIRPα Signal-regulatory protein alpha; SLA, swine leukocyte antigen; SMPDL-3b, Sphingomyelin phosphodiesterase acid-like 3b; Tg, Transgenic; TSP-1, thrombospondin-1; UAB, University of Alabama; UMMC, University of Maryland Medical Center; XTx, Xenotransplantation; VTL, Vascularized thymic lobe.

## References

[1] Hume DM Merrill JP Miller BF Thorn GW. Experiences With Renal Homotransplantation in the Human: Report of Nine Cases. J Clin Invest (1955) 34:327–82. doi: 10.1172/JCI103085 PMC438633 13233354

[2] Murray G. The Trend in Medicine. Am J Surg (1962) 103:155–6. doi: 10.1016/0002-9610(62)90478-6 14477457

[3] Cyclosporin and Neoplasia. Lancet (1983) 1:1083. 6133110

[4] Auchincloss H Jr. Xenogeneic Transplantation: A Review. Transplantation (1988) 46:1–20. doi: 10.1097/00007890-198807000-00001 3293272

[5] Auchincloss H Jr. Sachs DH. Xenogeneic Transplantation. Annu Rev Immunol (1998) 16:433–70. doi: 10.1146/annurev.immunol.16.1.433 9597137

[6] Buhler L Friedman T Iacomini J Cooper DK. Xenotransplantation–state of the Art–Update 1999. Front Biosci (1999) 4:D416–32. doi: 10.2741/Buhler 10209058

[7] Buhler L Yamada K Kitamura H Alwayn IP Basker M Appel JZ III. Pig Kidney Transplantation in Baboons: Anti-Gal(alpha)1-3gal IgM Alone is Associated With Acute Humoral Xenograft Rejection and Disseminated Intravascular Coagulation. Transplantation (2001) 72:1743–52. doi: 10.1097/00007890-200112150-00007 11740383

[8] Cooper DK. A Brief History of Cross-Species Organ Transplantation. Proc (Bayl Univ Med Cent) (2012) 25:49–57. doi: 10.1080/08998280.2012.11928783 22275786 PMC3246856

[9] Sachs DH. The Pig as a Potential Xenograft Donor. Vet Immunol Immunopathol (1994) 43:185–91. doi: 10.1016/0165-2427(94)90135-X 7856051

[10] Sachs DH Galli C. Genetic Manipulation in Pigs. Curr Opin Organ Transplant (2009) 14:148–53. doi: 10.1097/MOT.0b013e3283292549 PMC2687522 19469029

[11] Sachs DH Sykes M Robson SC Cooper DK. Xenotransplantation. Adv Immunol (2001) 79:129–223. doi: 10.1016/S0065-2776(01)79004-9 11680007

[12] Patience C Takeuchi Y Weiss RA. Infection of Human Cells by an Endogenous Retrovirus of Pigs. Nat Med (1997) 3:282–6. doi: 10.1038/nm0397-282 9055854

[13] Niu D Wei HJ Lin L George H Wang T Lee IH. Inactivation of Porcine Endogenous Retrovirus in Pigs Using CRISPR-Cas9. Science (2017) 357:1303–7. doi: 10.1126/science.aan4187 PMC5813284 28798043

[14] Fishman JA. Assessment of Infectious Risk in Clinical Xenotransplantation: The Lessons for Clinical Allotransplantation. Xenotransplantation (2014) 21:307–8. doi: 10.1111/xen.12118 PMC7169700 25098837

[15] Cowan PJ Tector AJ. The Resurgence of Xenotransplantation. Am J Transplant (2017) 17:2531–6. doi: 10.1111/ajt.14311 28397351

[16] Cooper DKC Hara H Iwase H Yamamoto T Li Q Ezzelarab M. Justification of Specific Genetic Modifications in Pigs for Clinical Organ Xenotransplantation. Xenotransplantation (2019) 26:e12516. doi: 10.1111/xen.12516 30989742 PMC10154075

[17] Iwase H Hara H Ezzelarab M Li T Zhang Z Gao B. Immunological and Physiological Observations in Baboons With Life-Supporting Genetically Engineered Pig Kidney Grafts. Xenotransplantation (2017) 24(2). doi: 10.1111/xen.12293 PMC5397334 28303661

[18] Rivard CJ Tanabe T Lanaspa MA Watanabe H Nomura S Andres-Hernando A. Upregulation of CD80 on Glomerular Podocytes Plays an Important Role in Development of Proteinuria Following Pig-to-Baboon Xeno-Renal Transplantation - an Experimental Study. Transpl Int (2018) 31:1164–77. doi: 10.1111/tri.13273 PMC6407427 29722117

[19] Langin M Mayr T Reichart B Michel S Buchholz S Guethoff S. Consistent Success in Life-Supporting Porcine Cardiac Xenotransplantation. Nature (2018) 564:430–3. doi: 10.1038/s41586-018-0765-z 30518863

[20] Mohiuddin MM Singh AK Corcoran PC Hoyt RF Thomas ML III Lewis BG. One-Year Heterotopic Cardiac Xenograft Survival in a Pig to Baboon Model. Am J Transplant (2014) 14:488–9. doi: 10.1111/ajt.12562 PMC4184155 24330419

[21] Mohiuddin MM Singh AK Corcoran PC Thomas ML 3rd Clark T Lewis BG. Chimeric 2C10R4 Anti-CD40 Antibody Therapy is Critical for Long-Term Survival of GTKO.hCD46.hTBM Pig-to-Primate Cardiac Xenograft. Nat Commun (2016) 7:11138. doi: 10.1038/ncomms11138 27045379 PMC4822024

[22] Kim SC Mathews DV Breeden CP Higginbotham LB Ladowski J Martens G. Long-Term Survival of Pig-to-Rhesus Macaque Renal Xenografts is Dependent on CD4 T Cell Depletion. Am J Transplant (2019) 19:2174–85. doi: 10.1111/ajt.15329 PMC6658347 30821922

[23] Galili U Rachmilewitz EA Peleg A Flechner I. A Unique Natural Human IgG Antibody With Anti-Alpha-Galactosyl Specificity. J Exp Med (1984) 160:1519–31. doi: 10.1084/jem.160.5.1519 PMC2187506 6491603

[24] Galili U Mandrell RE Hamadeh RM Shohet SB Griffiss JM. Interaction Between Human Natural Anti-Alpha-Galactosyl Immunoglobulin G and Bacteria of the Human Flora. Infect Immun (1988) 56:1730–7. doi: 10.1128/iai.56.7.1730-1737.1988 PMC259469 3290105

[25] Kolber-Simonds D Lai L Watt SR Denaro M Arn S Augenstein ML. Production of a-1,3-Galactosyltransferase Null Pigs by Means of Nuclear Transfer With Fibroblasts Bearing Loss of Heterozygosity Mutations. Proc Natl Acad Sci U S A (2004) 101:7335–40. doi: 10.1073/pnas.0307819101 PMC409919 15123792

[26] Lai L Kolber-Simonds D Park K Cheong H Greenstein JL Im G. Production of a-1,3-Galactosyltransferase Knockout Pigs by Nuclear Transfer Cloning. Science (2002) 295:1089–92. doi: 10.1126/science.1068228 11778012

[27] Dai Y Vaught TD Boone J Chen SH Phelps CJ Ball S. Targeted Disruption of the Alpha1,3-Galactosyltransferase Gene in Cloned Pigs. Nat Biotechnol (2002) 20:251–5. doi: 10.1038/nbt0302-251 11875425

[28] Varki A. Colloquium Paper: Uniquely Human Evolution of Sialic Acid Genetics and Biology. Proc Natl Acad Sci USA (2010) 107 Suppl 2:8939–46. doi: 10.1073/pnas.0914634107 PMC3024026 20445087

[29] Byrne GW Du Z Stalboerger P Kogelberg H McGregor CG. Cloning and Expression of Porcine Beta1,4 N-Acetylgalactosaminyl Transferase Encoding a New Xenoreactive Antigen. Xenotransplantation (2014) 21:543–54. doi: 10.1111/xen.12124 PMC4262693 25176027

[30] Byrne G Ahmad-Villiers S Du Z McGregor C. B4GALNT2 and Xenotransplantation: A Newly Appreciated Xenogeneic Antigen. Xenotransplantation (2018) 25:e12394. doi: 10.1111/xen.12394 29604134 PMC6158069

[31] Martens GR Reyes LM Li P Butler JR Ladowski JM Estrada JL. Humoral Reactivity of Renal Transplant-Waitlisted Patients to Cells From GGTA1/CMAH/B4GalNT2, and SLA Class I Knockout Pigs. Transplantation (2017) 101:e86–92. doi: 10.1097/TP.0000000000001646 PMC7228580 28114170

[32] Dalmasso AP. The Complement System in Xenotransplantation. Immunopharmacology (1992) 24:149–60. doi: 10.1016/0162-3109(92)90020-D 1473965

[33] Cozzi E White DJ. The Generation of Transgenic Pigs as Potential Organ Donors for Humans. Nat Med (1995) 1:964–6. doi: 10.1038/nm0995-964 7585226

[34] Lin Y Vandeputte M Waer M. Natural Killer Cell- and Macrophage-Mediated Rejection of Concordant Xenografts in the Absence of T and B Cell Responses. J Immunol (1997) 158:5658–67. 9190914

[35] Puga Yung G Schneider MKJ Seebach JD. The Role of NK Cells in Pig-To-Human Xenotransplantation. J Immunol Res (2017) 2017:4627384. 29410970 10.1155/2017/4627384 PMC5749293

[36] Ide K Wang H Tahara H Liu J Wang X Asahara T. Role for CD47-SIRPalpha Signaling in Xenograft Rejection by Macrophages. Proc Natl Acad Sci USA (2007) 104:5062–6. doi: 10.1073/pnas.0609661104 PMC1829264 17360380

[37] Moses RD Pierson RN 3rd Winn HJ Auchincloss H Jr. Xenogeneic Proliferation and Lymphokine Production are Dependent on CD4+ Helper T Cells and Self Antigen-Presenting Cells in the Mouse. J Exp Med (1990) 172:567–75. doi: 10.1084/jem.172.2.567 PMC2188348 2142721

[38] Moses RD Winn HJ Auchincloss H Jr. Evidence That Multiple Defects in Cell-Surface Molecule Interactions Across Species Differences are Responsible for Diminished Xenogeneic T Cell Responses. Transplantation (1992) 53:203–9. doi: 10.1097/00007890-199201000-00039 1346345

[39] Yamada K Sachs DH DerSimonian H. Human Anti-Porcine Xenogeneic T-Cell Response. Evidence for Allelic Specificity of MLR and for Both Direct and Indirect Pathways of Recognition. J Immunol (1995) 155:5249–56. 7594537

[40] Kalscheuer H Onoe T Dahmani A Li HW Holzl M Yamada K. Xenograft Tolerance and Immune Function of Human T Cells Developing in Pig Thymus Xenografts. J Immunol (2014) 192:3442–50. doi: 10.4049/jimmunol.1302886 PMC3983999 24591363

[41] Dorling A Lombardi G Binns R Lechler RI. Detection of Primary Direct and Indirect Human Anti-Porcine T Cell Responses Using a Porcine Dendritic Cell Population. Eur J Immunol (1996) 26:1378–87. doi: 10.1002/eji.1830260630 8647220

[42] Galili U. Induced Anti-non Gal Antibodies in Human Xenograft Recipients. Transplantation (2012) 93:11–6. doi: 10.1097/TP.0b013e31823be870 22146315

[43] Barth RN Yamamoto S LaMattina JC Kumagai N Kitamura H Vagefi PA. Xenogeneic Thymokidney and Thymic Tissue Transplantation in a Pig-to-Baboon Model: I. Evidence for Pig-Specific T-Cell Unresponsiveness. Transplantation (2003) 75:1615–24. doi: 10.1097/01.TP.0000064335.50622.20 12777846

[44] Yamada K Yazawa K Shimizu A Iwanaga T Hisashi Y Nuhn M. Marked Prolongation of Porcine Renal Xenograft Survival in Baboons Through the Use of Alpha1,3-Galactosyltransferase Gene-Knockout Donors and the Cotransplantation of Vascularized Thymic Tissue. Nat Med (2005) 11:32–4. doi: 10.1038/nm1172 15619627

[45] Pierson RN 3rd. Antibody-Mediated Xenograft Injury: Mechanisms and Protective Strategies. Transpl Immunol (2009) 21:65–9. doi: 10.1016/j.trim.2009.03.008 PMC2695451 19376229

[46] McCurry KR Kooyman DL Alvarado CG Cotterell AH Martin MJ Logan JS. Human Complement Regulatory Proteins Protect Swine-to-Primate Cardiac Xenografts From Humoral Injury. Nat Med (1995) 1:423–7. doi: 10.1038/nm0595-423 7585088

[47] Kolber-Simonds D Lai L Watt SR Denaro M Arn S Augenstein ML. Production of Alpha-1,3-Galactosyltransferase Null Pigs by Means of Nuclear Transfer With Fibroblasts Bearing Loss of Heterozygosity Mutations. Proc Natl Acad Sci USA (2004) 101:7335–40. doi: 10.1073/pnas.0307819101 PMC409919 15123792

[48] Kuwaki K Tseng YL Dor FJ Shimizu A Houser SL Sanderson TM. Heart Transplantation in Baboons Using Alpha1,3-Galactosyltransferase Gene-Knockout Pigs as Donors: Initial Experience. Nat Med (2005) 11:29–31. doi: 10.1038/nm1171 15619628

[49] Bouhours D Pourcel C Bouhours JE. Simultaneous Expression by Porcine Aorta Endothelial Cells of Glycosphingolipids Bearing the Major Epitope for Human Xenoreactive Antibodies (Gal Alpha 1-3Gal), Blood Group H Determinant and N-Glycolylneuraminic Acid. Glycoconj J (1996) 13:947–53. doi: 10.1007/BF01053190 8981086

[50] Padler-Karavani V Varki A. Potential Impact of the non-Human Sialic Acid N-Glycolylneuraminic Acid on Transplant Rejection Risk. Xenotransplantation (2011) 18:1–5. doi: 10.1111/j.1399-3089.2011.00622.x 21342282 PMC3098739

[51] Ma D Hirose T Lassiter G Sasaki H Rosales I Coe TM. Kidney Transplantation From Triple-Knockout Pigs Expressing Multiple Human Proteins in Cynomolgus Macaques. Am J Transplant (2022) 22:46–57. doi: 10.1111/ajt.16780 34331749 PMC9291868

[52] Estrada JL Martens G Li P Adams A Newell KA Ford ML. Evaluation of Human and non-Human Primate Antibody Binding to Pig Cells Lacking GGTA1/CMAH/beta4GalNT2 Genes. Xenotransplantation (2015) 22:194–202. doi: 10.1111/xen.12161 25728481 PMC4464961

[53] Diamond LE Quinn CM Martin MJ Lawson J Platt JL Logan JS. A Human CD46 Transgenic Pig Model System for the Study of Discordant Xenotransplantation. Transplantation (2001) 71:132–42. doi: 10.1097/00007890-200101150-00021 11211178

[54] Pierson RN 3rd Dorling A Ayares D Rees MA Seebach JD Fishman JA. Current Status of Xenotransplantation and Prospects for Clinical Application. Xenotransplantation (2009) 16:263–80. doi: 10.1111/j.1399-3089.2009.00534.x PMC2866107 19796067

[55] Petersen B Ramackers W Lucas-Hahn A Lemme E Hassel P Queisser AL. Transgenic Expression of Human Heme Oxygenase-1 in Pigs Confers Resistance Against Xenograft Rejection During *Ex Vivo* Perfusion of Porcine Kidneys. Xenotransplantation (2011) 18:355–68. doi: 10.1111/j.1399-3089.2011.00674.x 22168142

[56] Yeom HJ Koo OJ Yang J Cho B Hwang JI Park SJ. Generation and Characterization of Human Heme Oxygenase-1 Transgenic Pigs. PloS One (2012) 7:e46646. doi: 10.1371/journal.pone.0046646 23071605 PMC3465346

[57] Tena A Kurtz J Leonard DA Dobrinsky JR Terlouw SL Mtango N. Transgenic Expression of Human CD47 Markedly Increases Engraftment in a Murine Model of Pig-to-Human Hematopoietic Cell Transplantation. Am J Transplant (2014) 14:2713–22. doi: 10.1111/ajt.12918 PMC4236244 25278264

[58] Ariyoshi Y Takeuchi K Pomposelli T Ekanayake-Alper DK Shimizu A Boyd L. Antibody Reactivity With New Antigens Revealed in Multi-Transgenic Triple Knockout Pigs may Cause Early Loss of Pig Kidneys in Baboons. Xenotransplantation (2021) 28:e12642. doi: 10.1111/xen.12642 32909301 PMC8957702

[59] Takeuchi K Ariyoshi Y Shimizu A Okumura Y Cara-Fuentes G Garcia GE. Expression of Human CD47 in Pig Glomeruli Prevents Proteinuria and Prolongs Graft Survival Following Pig-to-Baboon Xenotransplantation. Xenotransplantation (2021) 28:e12708. doi: 10.1111/xen.12708 34418164 PMC8957703

[60] Murray AG Khodadoust MM Pober JS Bothwell AL. Porcine Aortic Endothelial Cells Activate Human T Cells: Direct Presentation of MHC Antigens and Costimulation by Ligands for Human CD2 and CD28. Immunity (1994) 1:57–63. doi: 10.1016/1074-7613(94)90009-4 7889399

[61] Zhao Y Swenson K Sergio JJ Arn JS Sachs DH Sykes M. Skin Graft Tolerance Across a Discordant Xenogeneic Barrier. Nat Med (1996) 2:1211–6. doi: 10.1038/nm1196-1211 8898747

[62] Rodriguez-Barbosa JI Zhao Y Barth R Zhao G Arn JS Sachs DH. Enhanced CD4 Reconstitution by Grafting Neonatal Porcine Tissue in Alternative Locations is Associated With Donor-Specific Tolerance and Suppression of Preexisting Xenoreactive T Cells. Transplantation (2001) 72:1223–31. doi: 10.1097/00007890-200110150-00007 11602846

[63] Haller GW Esnaola N Yamada K Wu A Shimizu A Hansen A. Thymic Transplantation Across an MHC Class I Barrier in Swine. J Immunol (1999) 163:3785–92. 10490976

[64] Yamada K Shimizu A Ierino FL Utsugi R Barth R Esnaola N. Thymic Transplantation in Miniature Swine. I. Development and Function of the "Thymokidney". Transplantation (1999) 68:1684–92. doi: 10.1097/00007890-199912150-00011 10609944

[65] LaMattina JC Kumagai N Barth RN Yamamoto S Kitamura H Moran SG. Vascularized Thymic Lobe Transplantation in Miniature Swine: I. Vascularized Thymic Lobe Allografts Support Thymopoiesis. Transplantation (2002) 73:826–31. doi: 10.1097/00007890-200203150-00032 11907438

[66] Yamada K Shimizu A Utsugi R Ierino FL Gargollo P Haller GW. Thymic Transplantation in Miniature Swine. II. Induction of Tolerance by Transplantation of Composite Thymokidneys to Thymectomized Recipients. J Immunol (2000) 164:3079–86. doi: 10.4049/jimmunol.164.6.3079 10706697

[67] Yamada K Vagefi PA Utsugi R Kitamura H Barth RN LaMattina JC. Thymic Transplantation in Miniature Swine: III. Induction of Tolerance by Transplantation of Composite Thymokidneys Across Fully Major Histocompatibility Complex-Mismatched Barriers. Transplantation (2003) 76:530–6. doi: 10.1097/01.TP.0000080608.42480.E8 12923439

[68] Kamano C Vagefi PA Kumagai N Yamamoto S Barth RN LaMattina JC. Vascularized Thymic Lobe Transplantation in Miniature Swine: Thymopoiesis and Tolerance Induction Across Fully MHC-Mismatched Barriers. Proc Natl Acad Sci USA (2004) 101:3827–32. doi: 10.1073/pnas.0306666101 PMC374329 15007168

[69] Nobori S Shimizu A Okumi M Samelson-Jones E Griesemer A Hirakata A. Thymic Rejuvenation and the Induction of Tolerance by Adult Thymic Grafts. Proc Natl Acad Sci USA (2006) 103:19081–6. doi: 10.1073/pnas.0605159103 PMC1748180 17148614

[70] Nobori S Samelson-Jones E Shimizu A Hisashi Y Yamamoto S Kamano C. Long-Term Acceptance of Fully Allogeneic Cardiac Grafts by Cotransplantation of Vascularized Thymus in Miniature Swine. Transplantation (2006) 81:26–35. doi: 10.1097/01.tp.0000200368.03991.e0 16421473

[71] Tasaki M Shimizu A Hanekamp I Torabi R Villani V Yamada K. Rituximab Treatment Prevents the Early Development of Proteinuria Following Pig-to-Baboon Xeno-Kidney Transplantation. J Am Soc Nephrol (2014) 25:737–44. doi: 10.1681/ASN.2013040363 PMC3968493 24459229

[72] Nomura S Ariyoshi Y Watanabe H Pomposelli T Takeuchi K Garcia G. Transgenic Expression of Human CD47 Reduces Phagocytosis of Porcine Endothelial Cells and Podocytes by Baboon and Human Macrophages. Xenotransplantation (2019):e12549.doi: 10.1111/xen.12549. 31495971 PMC7007337

[73] Sachs DH. Mixed Chimerism as an Approach to Transplantation Tolerance. Clin Immunol (2000) 95:S63–8. doi: 10.1006/clim.1999.4814 10729238

[74] Sykes M. Mechanisms of Tolerance Induced *via* Mixed Chimerism. Front Biosci (2007) 12:2922–34. doi: 10.2741/2282 17485269

[75] Sykes M. Hematopoietic Cell Transplantation for the Induction of Allo- and Xenotolerance. Clin Transplant (1996) 10:357–63. 8884109

[76] Kawai T Sachs DH Sykes M Cosimi AB. HLA-Mismatched Renal Transplantation Without Maintenance Immunosuppression. N Engl J Med (2013) 368:1850–2. doi: 10.1056/NEJMc1213779 PMC3760499 23656665

[77] Leventhal J Abecassis M Miller J Gallon L Tollerud D Elliott MJ. Tolerance Induction in HLA Disparate Living Donor Kidney Transplantation by Donor Stem Cell Infusion: Durable Chimerism Predicts Outcome. Transplantation (2013) 95:169–76. doi: 10.1097/TP.0b013e3182782fc1 PMC3531567 23222893

[78] Scandling JD Busque S Shizuru JA Lowsky R Hoppe R Dejbakhsh-Jones S. Chimerism, Graft Survival, and Withdrawal of Immunosuppressive Drugs in HLA Matched and Mismatched Patients After Living Donor Kidney and Hematopoietic Cell Transplantation. Am J Transplant (2015) 15:695–704. doi: 10.1111/ajt.13091 25693475

[79] Sharabi Y Aksentijevich I Sundt TM 3rd Sachs DH Sykes M. Specific Tolerance Induction Across a Xenogeneic Barrier: Production of Mixed Rat/Mouse Lymphohematopoietic Chimeras Using a Nonlethal Preparative Regimen. J Exp Med (1990) 172:195–202. doi: 10.1084/jem.172.1.195 1972728 PMC2188183

[80] Yang YG DeGoma E Ohdan H Bracy JL Xu YX Iacomini J. Tolerization of Anti-Gala1-3Gal Natural Antibody-Forming B Cells by Induction of Mixed Chimerism. J Exp Med (1998) 187:1335–42. doi: 10.1084/jem.187.8.1335 PMC2212239 9547344

[81] Ohdan H Yang YG Shimizu A Swenson KG Sykes M. Mixed Chimerism Induced Without Lethal Conditioning Prevents T Cell- and Anti-Gal Alpha 1,3Gal-Mediated Graft Rejection. J Clin Invest (1999) 104:281–90. doi: 10.1172/JCI6656 PMC408419 10430609

[82] Griesemer A Liang F Hirakata A Hirsh E Lo D Okumi M. Occurrence of Specific Humoral non-Responsiveness to Swine Antigens Following Administration of GalT-KO Bone Marrow to Baboons. Xenotransplantation (2010) 17:300–12. doi: 10.1111/j.1399-3089.2010.00600.x PMC2942069 20723202

[83] Liang F Wamala I Scalea J Tena A Cormack T Pratts S. Increased Levels of Anti-non-Gal IgG Following Pig-to-Baboon Bone Marrow Transplantation Correlate With Failure of Engraftment. Xenotransplantation (2013) 20:458–68. doi: 10.1111/xen.12065 PMC3848062 24289469

[84] Ide K Ohdan H Kobayashi T Hara H Ishiyama K Asahara T. Antibody- and Complement-Independent Phagocytotic and Cytolytic Activities of Human Macrophages Toward Porcine Cells. Xenotransplantation (2005) 12:181–8. doi: 10.1111/j.1399-3089.2005.00222.x 15807768

[85] Wang H VerHalen J Madariaga ML Xiang S Wang S Lan P. Attenuation of Phagocytosis of Xenogeneic Cells by Manipulating CD47. Blood (2007) 109:836–42. doi: 10.1182/blood-2006-04-019794 PMC1785095 17008545

[86] Tena AA Sachs DH Mallard C Yang YG Tasaki M Farkash E. Prolonged Survival of Pig Skin on Baboons After Administration of Pig Cells Expressing Human Cd47. Transplantation (2017) 101:316–21. doi: 10.1097/TP.0000000000001267 PMC5124423 27232934

[87] Tasaki M Wamala I Tena A Villani V Sekijima M Pathiraja V. High Incidence of Xenogenic Bone Marrow Engraftment in Pig-to-Baboon Intra-Bone Bone Marrow Transplantation. Am J Transplant (2015) 15:974–83. doi: 10.1111/ajt.13070 PMC4407988 25676635

[88] Watanabe H Ariyoshi Y Pomposelli T Takeuchi K Ekanayake-Alper DK Boyd LK. Intra-Bone Bone Marrow Transplantation From Hcd47 Transgenic Pigs to Baboons Prolongs Chimerism to >60 Days and Promotes Increased Porcine Lung Transplant Survival. Xenotransplantation (2020) 27:e12552. doi: 10.1111/xen.12552 31544995 PMC7007336

